# A 3-Week Tryptophan-Deficient Diet Resulted in Decreased Body Weight and Increased Trabecular Bone Mass in Mice

**DOI:** 10.1093/geroni/igaa057.403

**Published:** 2020-12-16

**Authors:** Carlos Isales, Kehong Ding, Meghan McGee-Lawrence, Wendy Bollag, William Hill, Sadanand Fulzele, Mohamed Awad, Mark Hamrick

**Affiliations:** 1 Augusta University, Augusta, Georgia, United States; 2 Medical University of South Carolina, Charleston, South Carolina, United States

## Abstract

Tryptophan is an essential amino acid with a variety of bioactive metabolites including serotonin, melatonin and nicotinamide. Dietary Trp restriction results in increased lifespan but with detrimental side effects. The initial catabolite in the Trp breakdown pathway, kynurenine, increases with aging and induces bone loss. Thus, we hypothesized that eliminating Trp in the diet of older mice might be osteoprotective. In an IACUC-approved protocol, we fed either 0, 0.2 (standard), 0.7 or 1.25% Trp-containing diets to aged (23-month-old) C57BL/6 mice for a planned eight weeks. There was a rapid decrease in body weight in the mice fed the 0% tryptophan diet and the mice had to be sacrificed at three weeks (25.2±1.4 vs 35.4±3.6 vs 33.9±2.4 vs 33.4±3.1 gm, (Means+SD, No Trp: 0%; Standard Trp 0.2%; High Trp: 0.7%; High Trp: 1.25%, p=0.0004, 0 vs 0.2%, n=9-10/group). Removal of Trp from the diet had a differential effect on bone mineral density (BMD). Total BMD was increased in the low Trp group: (0.0548±0.0017 vs 0.518±0.0021 vs 0.0526±0.0021 vs 0.0524±0.0022, Means+SD, No Trp: 0%; Standard Trp 0.2%; High Trp: 0.7%; High Trp: 1.25%, p=0.016, 0 vs 0.2%). Femoral BMD did not change; however, spinal BMD rose (0.0603±0.005 vs 0.0477±0.06 vs 0.0505±0.005 vs 0.0535±006; Means+SD, Trp 0% vs 0.2%, p=0.00009). The mice on the 0% Trp diet also had lower body fat (22.4±2.3% vs 26.7±3.3%; Means+SD, Trp 0 vs 0.2%, p=0.005). Thus, dietary Trp restriction resulted in a beneficial increase in spinal BMD, though at least in part related to weight loss.

